# Identifying the Impact of Intimate Partner Violence in Humanitarian Settings: Using an Ecological Framework to Review 15 Years of Evidence

**DOI:** 10.3390/ijerph18136963

**Published:** 2021-06-29

**Authors:** Melissa Meinhart, Ilana Seff, Katrina Troy, Samantha McNelly, Luissa Vahedi, Catherine Poulton, Lindsay Stark

**Affiliations:** 1 Brown School of Social Work, Washington University, in St. Louis 1 Brookings Drive, St. Louis, MO 63130, USA; melissa.m.meinhart@gmail.com (M.M.); seff@wustl.edu (I.S.); mskatrinatroy@gmail.com (K.T.); mcnelly.s@wustl.edu (S.M.); l.vahedi@wustl.edu (L.V.); 2 UNICEF, United Nations Plaza, New York, NY 10017, USA; cpoulton@unicef.org

**Keywords:** intimate partner violence, gender-based violence, humanitarian settings, ecological frameworks, systematic review

## Abstract

Intimate partner violence (IPV) is a pervasive form of gender-based violence that exacerbates in humanitarian settings. This systematic review examined the myriad IPV impacts and the quality of existing evidence of IPV in humanitarian settings. Following the Preferred Reporting Items for Systematic Reviews and Meta-Analyses (PRISMA) procedures, a total of 51 articles were included from the 3924 screened. We identified the impact of IPV across two levels of the ecological framework: individual and microsystem. Our findings corroborated previous evidence that indicated IPV to be associated with adverse physical and mental health for survivors. Our findings also uniquely synthesized the intergenerational impact of IPV in humanitarian settings. However, findings highlighted a glaring gap in evidence examining the non-health impact of IPV for survivors in humanitarian settings and across levels of the ecological framework. Without enhanced research of women and girls and the violence they experience, humanitarian responses will continue to underachieve, and the needs of women and girls will continue to be relegated as secondary interests. Investment should prioritize addressing the range of both health and non-health impacts of IPV among individuals, families, and communities, as well as consider how the humanitarian environment influences these linkages.

## 1. Introduction

Intimate Partner Violence (IPV) is a critically concerning form of gender-based violence in humanitarian settings. IPV includes violence that occurs within an intimate relationship of romantic partners or ex-partners, whether cohabitating or not [1]. Moreover, IPV can be exhibited through physical, sexual, or psychological harm and includes physical aggression, sexual coercion, psychological abuse, and controlling behaviors [2]. Violence perpetrated by intimate partners within the private sphere of the home continues to be an insidious form of gender-based violence during periods of acute crisis as well as protracted emergencies and post-conflict settings [3,4,5,6,7]. In humanitarian settings, known predictors of interpersonal violence within the household, including IPV, include conflict exposure, substance use, low economic status, adverse mental health, and limited social support [8], and many known determinants of IPV are exacerbated in emergency settings [9].

While less is known regarding the widespread impacts of IPV in humanitarian settings specifically, several reviews have synthesized evidence examining the global impacts of IPV on survivors. One review, not specific to low- and middle-income countries (LMICs) nor humanitarian settings, identified consistent evidence that IPV affected sexual risk-taking behaviors, risk of lifetime sexually transmitted infections (STIs), unwanted pregnancy or induced abortion, and sexual dysfunction [10]. Another review examining both physical and mental health consequences found that IPV survivors were more likely to exhibit physical injury, chronic pain, gastrointestinal issues, gynecological disorders, depression, and post-traumatic stress disorder (PTSD) [11]. Systematic reviews of violence against women and girls in LMICs found that IPV was associated with composite measures of adverse mental health, suicidal ideation and behavior, and symptoms of depression, posttraumatic stress, and disordered eating [12], as well as STIs, unwanted pregnancy or induced abortion, and number of sexual partners [13]. While these four reviews bolster our understanding of the consequences of experiencing IPV, they have three notable gaps: (1) these reviews did not consider IPV in humanitarian settings, (2) these reviews did not include non-health correlates with IPV, and (3) these reviews did not examine the impacts of IPV beyond the survivor. The importance of addressing each of these gaps is described below.

Humanitarian crises are associated with periods of extreme chronic stress, loss of health and social service infrastructure, and strained social support networks; all can serve to exacerbate the impacts of IPV [14,15]. Moreover, this fundamental breakdown within humanitarian settings could contribute to unique consequences of IPV when compared to non-humanitarian settings. For example, the socioeconomic precarity within humanitarian settings may not only contribute to elevated prevalence of IPV but may also limit survivors’ abilities to return to or search for employment after experiencing IPV. Data collection of IPV in humanitarian settings is also difficult due to the instability inherent to natural disasters, infectious disease outbreaks, mass displacement, and civil unrest [6,16,17]. These measurement challenges have contributed to a paucity of IPV research in humanitarian settings; thus, little is known about how IPV impacts compare to those encountered in non-humanitarian settings.

Survivors in humanitarian settings may experience different types and severity of health impacts given the contextual variances between humanitarian and non-humanitarian settings. For example, women who would have otherwise accessed healthcare facilities for physical injuries from IPV may be unable to do so during a natural disaster, thus, increasing their risk for secondary infection or long-term disability. Moreover, research has yet to synthesize the non-health related impacts of IPV for survivors despite a growing body of evidence that has highlighted adverse economic outcomes for IPV survivors [18,19,20,21]. Other potential non-health impacts of IPV for survivors include loss of education and opportunity, productivity loss at work and home, stigma and shame, as well as diminished social capital, autonomy, and decision making.

Given the burden of violence placed on survivors, individual-level consequences of IPV are important to identify; however, IPV also has implications for the family unit and society. For example, witnessing IPV as a child has been linked to adverse mental health as well as an increased likelihood of perpetrating and experiencing IPV later in life [22,23,24]. IPV can also have harder-to-measure consequences at the community and societal levels. Women and girls who have experienced IPV may become further embedded in a cycle of victimization that perpetuates the feminization of poverty and the wider erosion of women’s sexual and reproductive agency. Gender-based violence, including IPV, has also been demonstrated to have harmful macroeconomic impacts in non-humanitarian settings [25,26,27,28,29,30]. Other potential impacts across the ecological framework include intra-household tensions and poverty, increased service needs and strains (health, legal, housing, justice, etc.), as well as perpetuation of harmful social norms, structural violence, and gender inequities.

**Figure 1:**
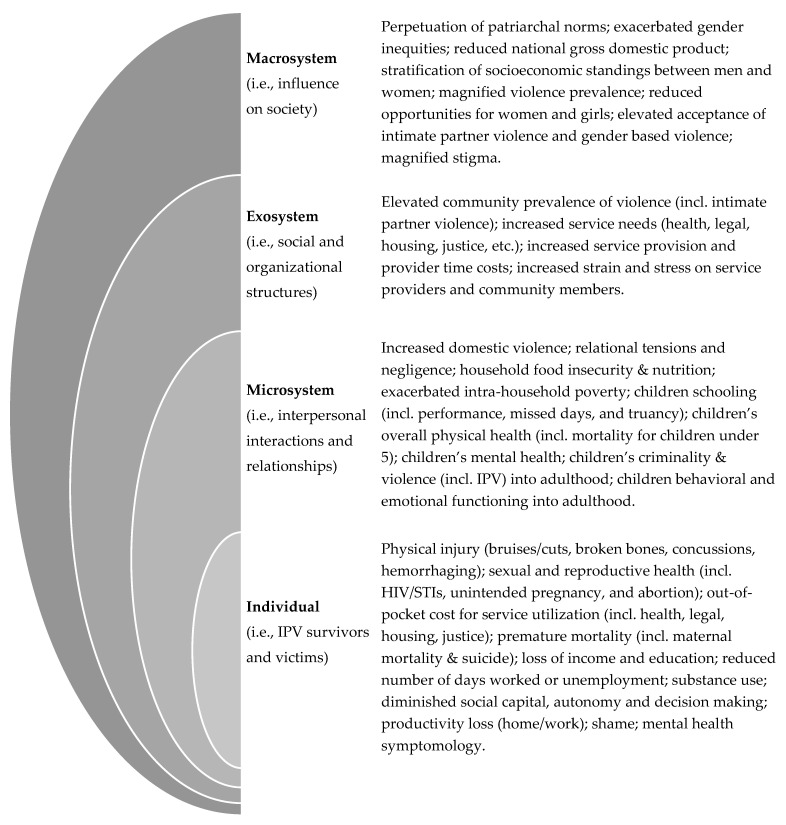
Impacts of intimate partner violence in humanitarian settings using an amended ecological framework.

Thus, it is critically important to examine the breadth of IPV impacts in humanitarian settings to understand the complexity of IPV and inform response efforts. Comprehensively examining IPV can enable the critical examination across and between all levels of society. Heise’s [31] seminal ecological framework illustrated the synergies between personal, situational, and sociocultural risk factors of male-perpetrated IPV across four levels: individual (i.e., IPV survivors and victims), microsystem (i.e., interpersonal interactions and relationships), exosystem (i.e., social and organizational structures), and macrosystem (i.e., influence on society). This risk framework has been adapted to humanitarian settings [9] and integrated for program design [32]; however, it has not been adapted as an impact framework. Building from the IPV and humanitarian literature, we adapted Heise’s ecological framework to hypothesize the myriad impacts of IPV in humanitarian settings. The adapted framework in Figure 1 guided the expansive interest of this systematic review by mapping potential impacts of IPV in humanitarian settings at the individual, microsystem, exosystem, and macrosystem levels.

Using the adapted ecological framework, this systematic review endeavored to examine the impact of IPV for survivors, families, communities, and society. Given the methodological challenges of collecting data in humanitarian settings, this review also examined the quality of studies from which the evidence was drawn. Understanding the full range of impacts that IPV has in humanitarian settings can simultaneously support critical programming for survivors and address the needs of families and communities affected by IPV.

## 2. Methods

### 2.1. Search Strategy

**Figure 2:**
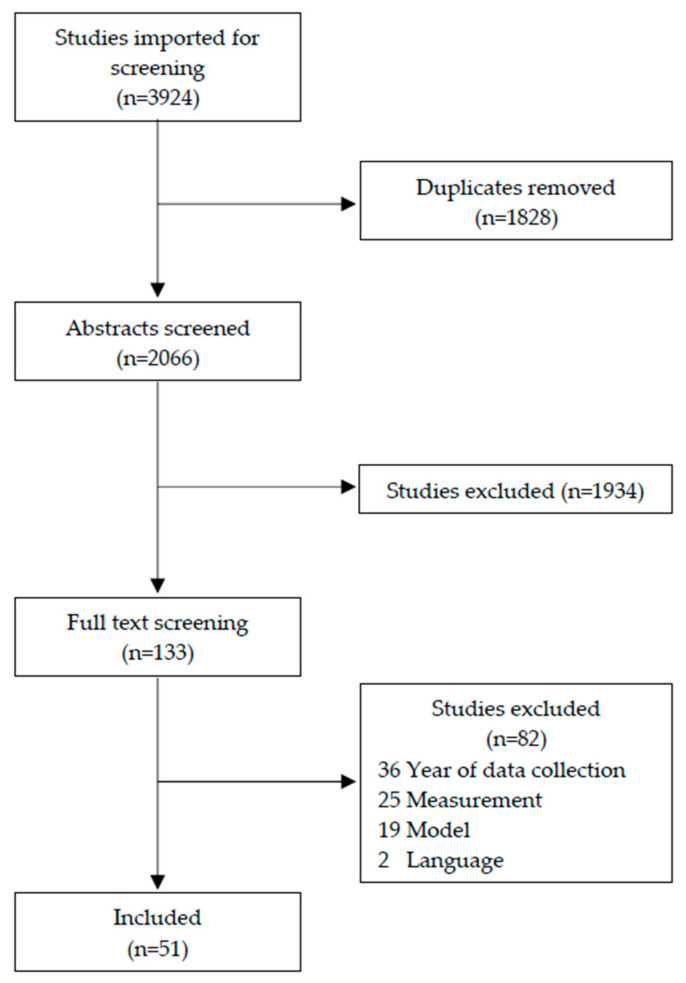
PRISMA flow diagram.

We conducted a systematic review of peer-reviewed articles that examined the impacts of IPV in humanitarian settings using the Preferred Reporting Items for Systematic Reviews and Meta-Analyses (PRISMA) guidelines [33]. Aligning with standardized violence definitions brokered by the WHO Multi-Country Study in 2005 on Women’s Health and Domestic Violence against Women [34], the search included articles published between 2005 and 2020. Our search terms strategy was applied to four databases: Embase through Elsevier, Medline via EBSCO, PsycInfo through Ebscohost, and Scopus via Scopus. Abstracts from the identified articles (N = 3924) were imported into Covidence for duplication removal, abstract review, and full-text review. All abstracts and full texts were reviewed by the authors to determine if they met review criteria at each stage. Conflicts at both the abstract and full-text review stages were reread and discussed by the authors for the final decision. A visual representation of the review process is available in Figure 2.

### 2.2. Inclusion and Exclusion Criteria

Using systematic review software (Covidence), abstracts were reviewed per inclusion criteria. Criteria included abstracts that utilized quantitative methods and abstracts that included at least one form of IPV as an independent variable. Examples of eligible IPV variables included experiencing physical IPV during adulthood, mean community rates of IPV, and witnessing intra-parental violence during childhood. Using the United Nation’s Financial Tracking System (FTS) [35], articles were considered eligible if their abstract mentioned that data were collected during at least one year when the country received humanitarian funding through either the Consolidated Appeals Process (CAP) or Humanitarian Response Planning (HRP). If the year of data collection was unclear, abstracts were flagged but included in the full-text review (n = 54). Countries that received flash and regional appeals were only included if they also received either direct CAP or HRP funding. Systematic reviews and dissertations were not included, but their reference lists were reviewed to identify potentially relevant articles.

The 133 articles that met the criteria for full-text review were closely examined to determine if the article met exclusion criteria. First, the authors checked to see if each article was available in English. Articles were then re-reviewed to ensure data collection overlapped at some point during receipt of CAP and HRP funding between 2005 and 2020. Articles that did not make explicit note of when data were collected were excluded. However, articles were included if there was any data collection during a CAP or HRP year; thus, some articles may include data that were collected during CAP/HRP and non-CAP/HRP years. Similarly, included articles may include some degree of data collection before 2005 if simultaneous data collection coincided with a CAP/HRP year. Articles were also more rigorously examined based on if their definition of IPV aligned with the Inter-Agency Standing Committee (IASC) guidelines definition [1]. For example, articles needed to either include the explicit IPV terminology or describe how their measure of violence—particularly interpersonal and domestic violence—was restricted to intimate partners. Finally, analytical models that did not disaggregate by country were excluded.

### 2.3. Data Extraction and Quality Assessment

Each article that met inclusion and exclusion criteria was re-reviewed in full (n = 51). Data extracted from each article related to the study’s sampling framework, variable creation, and analysis methods. Using the Appraisal tool for Cross-Sectional Studies (AXIS tool) [36], all included articles were individually scored based on 20 quality assessment criteria.

## 3. Findings

### 3.1. Overview of Included Articles

The majority of included articles collected data in sub-Saharan Africa (n = 33). The remaining articles, including those with data from multiple regions, included data from Central and Southeast Asia (n = 7), the Middle East (n = 7), or the Caribbean (n = 5). It is worth noting that all articles from the Caribbean were from Haiti and no articles were published from Central nor South America. Further, while 48 countries received CAP or HRP funding between 2005 and 2020, only 18 had at least one article that met inclusion criteria. While articles that included data collection from 2020 to 2018 were limited, the years of data collection were roughly equal across the remaining years 2005–2017 with an average of six articles including data collected from each of these years.

All of the articles included women and/or girls within the sample and five also included men and/or boys. The vast majority of articles utilized cross-sectional data either from a cross-sectional (72.55%, n = 37) or longitudinal study (17.65%, n = 9). Only 9.80% (n = 5) articles included analysis using longitudinal data. With 19.61% (n = 10) of articles using data from interventions, most of the remaining articles utilized population-based designs (41.18%, n = 21) or non-population-based designs (35.29%, n = 18). One article included data from an intervention study in Afghanistan and a non-population-based study design from Palestine. The final type of study design, a retrospective case review, was only used in one article that examined homicides in Maputo Province, Mozambique between 2016 and 2017.

The most common primary analysis model was multivariable logistic regression (60.78%, n = 31). Ten articles (19.61%) utilized other types of multivariable inferential modeling, including multivariable linear regression (9.80%, n = 5). One article included both multivariable linear and logistic regressions and another article utilized multivariable hierarchical regression. The primary models for the remaining nine articles that did not incorporate multivariable inferential modeling used either bivariate (13.73%, n = 7) or descriptive (3.92%, n = 2) analysis. See Table 1 for a comprehensive overview of the included articles.

### 3.2. Impacts of IPV

Consequences of IPV were identified across two primary levels: individual/survivor and microsystem/relationship (see Table 2). While all but six inferential articles demonstrated significant associations between at least one form of IPV and adverse individual-level impacts, these findings were often related to the ill-health of the survivor. Only two articles examined how direct IPV experience was associated with future IPV victimization (n = 1) or perpetration (n = 1), while five articles included variables related to healthcare access, healthcare adherence, or health status disclosure.

Except for one article that examined IPV perpetration and one article that examined IPV revictimization, all of the individual-level associations with IPV were directly related to ill-health (n = 41). The most common associations were in the mental health category (n = 11). Each of the 11 mental health studies included significant associations between IPV experience and worse mental health, with mental health variables ranging from aggregate wellness measures (including poor mental health and mental component summary) to specific conditions (including PTSD, substance abuse, and depression) or suicide variables (including ideation, thoughts, and attempts). The most common mental health variable was depression, which was included in six-articles and included perinatal depression, major depressive disorder, and past one-week, two-week, or four-week depression symptoms.

Other health categories at the individual level included the groupings of reproductive health (n = 8), HIV and other sexually transmitted infections (n = 7), pregnancy, birth, and infancy (n = 7), healthcare access, adherence, or disclosure (n = 5), overall health (n = 2), substance use (2.17% n = 1), and fatal injuries (n = 1). Significant findings were consistent across groupings, linking IPV with adverse physical health and mental health. Only seven of the articles included no significant results concerning individual-level impacts of IPV; moreover, there were two descriptive articles (n = 2) that were excluded when reviewing significance.

Fewer articles included impacts of IPV at the microsystem level (23.53%, n = 12). The 10 articles that examined the intergenerational impact of IPV between parents or caregivers often focused on IPV perpetration (n = 3) or victimization (n = 4) during adulthood. Other categories, each examined by one article, included physical health conditions during childhood, emotional and behavioral problems during childhood, and accepting attitudes of IPV during adulthood. All of these articles included significant associations with at least one of the IPV variable(s) and an adverse outcome, except one article that did not find a significant association between childhood exposure and perpetration during adulthood. Moreover, one article examined the significant association between IPV and marital disruption. The final article, examining the associations of IPV with relationships, identified that women who experienced physical violence in the past year were more likely to report a female relative being killed in the name of honor by another family member. While community-level IPV (i.e., societal level violence) was included as an independent variable in three articles, no articles examined societal-level impacts of IPV.

### 3.3. IPV Variables

The measurement of IPV varied across articles (See Table 3), with a little less than half including more than one measure of IPV (43.14%, n = 22). However, there were some notable themes in measurement, including the tendency to focus on direct victimization of violence (86.27%, n = 44). When measuring direct victimization of IPV, articles often included the timeframe of lifetime exposure for at least one of the violence variables (n = 29). The most common form of IPV was multiple or any form (n = 28), and most measures of direct victimization of IPV were binary (n = 37).

Fewer articles included variables that identified whether respondents witnessed IPV between their parents or primary caregivers during childhood (19.61%, n = 10). However, all of those articles included binary measurement of all forms of violence. Each of the articles (n = 3) that included a variable of community rates of IPV included multiple or any form of IPV; one article also included community rates of sexual violence. The timeframe used to determine community rates was either the past five-year reporting from secondary data (n = 1) or the aggregate lifetime experience of respondents in the sample (n = 2). The two measurement typologies for community violence were ordinal (n = 1) or ratio (n = 2).

### 3.4. Quality Assessment

As there are no formal AXIS scoring criteria, the authors ranked articles into four categories whereby scores of 15–20 were classified as the upper category, 10–14 as the upper-middle category, 5–9, as the lower-middle category, and 0–4 as the lowest category. Table 1 includes the quality assessment classification of each article and Table 4 includes the aggregate AXIS results per item. Nearly all articles met the criteria for the top 31.37% (n = 16) or upper-middle 62.75%% (n = 32) categories, with only three (5.88%) articles meeting the criteria for the lower-middle category and none meeting the criteria for the lowest category. The low overall scoring of items 13 and 14, both of which pertained to non-response rates, was not surprising given that only 45.10% (n = 23) of articles described measures taken to categorize non-responders (item 7). The other low score was related to the measurement of risk factors and dependent variables (item 9), whereby only 25.49% (n = 13) of the articles fully described how the primary variables were derived from instruments or measurements that had been trialed, piloted, or previously published.

The averages of the first four domains in the AXIS tool—introduction, methods, results, discussion—were relatively similar; however, the final domain of “other” included two items with low averages. The first item of the “other” domain asked about funding sources or conflicts of interest that may affect the authors’ interpretation of the results, whereby only half of the articles (n = 28) did not have overt conflicts. It is important to note that the “don’t know” option was selected whenever funding information was not provided; thus, those votes did not contribute to the final count. Similarly, only 68.63% (n = 35) of the articles reported ethical approval; however, this only reflects ethical approval reported within the articles. Some studies may have acquired ethical approval, but the authors did not report it. In both instances, these findings are important in regard to flagging the inconsistent reporting of ethical information.

## 4. Discussion

This review endeavored to identify the common and unique impacts of IPV in humanitarian settings across the entire ecological framework; however, no included article examined the impacts of IPV at the exosystem or macrosystem levels. Thus, we synthesized the existing empirical evidence at the individual and microsystem levels. Our synthesis corroborated previous reviews that indicated IPV to be associated with adverse physical and mental health outcomes for survivors at the individual level [10,11,12,13] and identified that IPV in humanitarian settings has detrimental impacts on family members at the microsystem level. Critically, the included articles for this review did not elucidate humanitarian-unique impacts of IPV. This is concerning, as there are many characteristics germane to humanitarian settings that could both exacerbate the prevalence of IPV [6] and magnify the impact of IPV [9,88]. Without a comprehensive understanding of IPV’s influence across the ecological framework, important considerations for programming and policy may be overlooked and funding may continue to underserve women and girls in humanitarian settings [89]. As the evidence-base expands, we advocate for future efforts to consider how the ecological framework proposed in Figure 1 may be refined to comprehensively reflect the impacts of IPV in humanitarian settings. Few of the hypothesized impacts of IPV from Figure 1 were explored in the included articles and no novel impacts were included within the study designs of the reviewed literature, thus, highlighting huge and important data gaps.

A striking challenge with examining the included research to ascertain the impacts of IPV during humanitarian crises was the reliance on lifetime experience of IPV as a single binary variable. In addition to reflecting an oversimplification or potential measurement issue, this lifetime experience of IPV could have occurred outside the period when a context would be considered a humanitarian setting. Only ten articles included IPV experienced within the past 1 to 12 months, but it is important to flag that all of them reported a significant association between IPV and the impacts of IPV. The number of selected articles was too small to rigorously compare but this finding highlights that IPV experienced during a humanitarian crisis may be more consistently associated with adverse IPV impacts than lifetime exposure. Future research should include explicit consideration regarding how different experiences of violence before, during, and after a humanitarian crisis may uniquely influence survivors, families, and communities. Research should also explore the interactions of IPV impacts between levels of ecological framework. Such research would begin to elucidate how and to what extent various levels of the ecological framework have interactive and reinforcing influence on the impacts of IPV (also known as reciprocal determinism). This type of interconnected thinking could bolster program efficiency by supporting more targeted programming to address the most acutely influential impacts of IPV [90,91].

When examining the quality assessment results, the AXIS tool yielded favorable scores for the included articles; however, there are still important gaps regarding the broader scientific evidence available. The choice of the AXIS tool was, in part, due to the cross-sectional nature of most articles. Only five of included articles analyzed longitudinal data, aligning with other reviews in non-humanitarian settings with a similar dearth in longitudinal data [12,13]. Longitudinal studies from humanitarian settings are needed to temporally understand the interplay of IPV experience, humanitarian conflict/emergency, and outcomes across the ecological model. Moreover, humanitarian IPV research is geographically stymied. Of the 48 countries that received either CAP or HRP funding between 2005 and 2020, data from only 18 countries (37.50%) were included within a publication that met our inclusion criteria. The vast majority of data were collected within the African continent, and there were no articles from South and Central America. The included studies were also limited in their ability to consider the temporal and geographical severity of humanitarian crises. Emergency designation (Level 1—Level 3) is available for current and recent crises [92], but there is no open-source designation of crisis severity between settings and across time. The INFORM Global Crisis Severity Index (GCSI) provides as an emerging initiative that may support examination of humanitarian crisis designation and IPV impacts for future research [93]. By integrating multi-level modeling techniques of GCSI designations or other secondary data sources like Armed Conflict Location and Event Data Project (ACLED) or Uppsala Conflict Data Program (UCDP) [4,54], researchers could examine the influence of various forms of IPV between crisis severity. In the meantime, researchers should consider operationalizing humanitarian stressors, such as self-reported conflict exposure and forced migration. Researchers and donors, alike, need to consider how to more inclusively and comprehensively fix these data gaps as they impede the ability to address the impacts of IPV in humanitarian settings.

## 5. Limitations

Limitations of this systematic review should be carefully considered. The overwhelmingly significant associations demonstrated in the findings may be a result of publication biases for articles that demonstrate significant findings. Thus, we cannot exhaustively state that other dependent variables have not been examined, only that other dependent variables were not available in the published peer-reviewed literature. Future research should examine and report on a range of outcomes that have been theorized to have associations with IPV. When including common impact variables, future research should build upon existing modelling to support comparability as the misalignment in measurement across studies hindered the possibility of meta-analysis for this systematic review. Finally, this systematic review utilized the AXIS tool for quality assessment despite not all articles using cross-sectional data. While the AXIS tool was developed for cross-sectional studies, each item within the AXIS tool was relevant for longitudinal research and the authors felt it was important to apply the same criteria for each article to enable comparability.

## 6. Conclusions

IPV in humanitarian settings is pervasive, and findings from this review indicated that its impacts are far-reaching. We demonstrated that IPV was significantly associated with a range of adverse health and non-health impacts for individuals and family members. However, there remains a paucity of inclusive research examining the novel impacts of IPV in humanitarian settings across the ecological framework. An understanding of the health and non-health impacts of IPV among survivors and, importantly, their families and communities, is critical for programming to thwart the widespread harms of IPV. With any hopes of achieving the Sustainable Development Goal of Gender Equality by 2030 [94], research and investment needs to prioritize the unique experiences of IPV by survivors in humanitarian settings and the impacts across the ecological framework.

## Figures and Tables

**Table 1:** Overview of included studies.

#	Citation	Year(s) of Data Collection	Country of Data Collection	Study Type	Study Name	Temporality	Primary Analysis Model	Outcome Variable(s) ^++++^	Quality Assessment
Middle East									
1	Goessmann, Ibrahim, and Neuner, 2020 [37]	2018–2019	Iraqi Kurdistan	Other non-population based design	Not stated	Cross-sectional	Multi-variable hierarchical regression	- Post-traumatic stress disorder - Depression	Top category
2	Al-Atrushi et al., 2013 [38]	2009–2011	Iraqi Kurdistan	Other non-population based design	Not stated	Cross-sectional	Descriptive analysis of women who reported lifetime IPV	- Any physical injury - Cuts, bruises, or aches - Eye injuries, sprain, dislocation, or burns - Stab wounds, broken bones, or broken teeth	Lower-middle category
3	Zakar et al., 2012 [39]	2008–2009	Pakistan	Other non-population based design	Not stated	Cross-sectional	Multi-variable logistic regression (forms of violence included within separate models)	- Past 6-mo poor reproductive health status	Top category
4	Zakar et al., 2013 [40]	2008–2009	Pakistan	Other non-population based design	Not stated	Cross-sectional	Multi-variable logistic regression (different forms of violence in separate models)	- Poor mental health	Top category
5	Sousa et al., 2018 [41]	2007–2008	Palestine	Other non-population based design	Not stated	Cross-sectional	Multi-variable regression (all forms of violence included within same model)	- Mental Component Summary (interval) - Distress (interval) - PTSD (interval)	Lower-middle category
6	Falb et al., 2019 [42]	2018	Syria	Intervention study	Not stated (cash transfer program)	Cross-sectional (baseline)	Multi-variable linear regression (all forms of violence included within same model)	- Past 2-week depression symptoms (ratio)	Top category
West Africa and the Sahel Region									
7	Fiorentino et al., 2019 [43]	2014	Cameroon	Other non-population based design	ANRS-12288 EVOLCam	Cross-sectional	Multi-variable logistic regression	- Recent ATI ≥ 1-month	Top category
8	Peltzer and Pengpid, 2014 [44]	2011–2012	Ivory Coast	Population-based design	Demographic Health Survey	Cross-sectional	Multi-variable logistic regression	- Lifetime any IPV - Lifetime physical IPV - Lifetime sexual IPV - Lifetime emotional IPV - Lifetime one type of IPV - Lifetime two or three types of IPV	Upper-middle category
9	Alenko, et al., 2020 [45]	2019	Ethiopia	Other non-population based design	Not stated	Cross-sectional	Multi-variable logistic regression	- Child emotional and behavioral problems	Top category
10	Gebreslasie et al., 2020 [46]	2017–2018	Ethiopia	Other non-population based design	Not stated	Cross-sectional	Multi-variable logistic regression	- Stillbirth	Top category
11	Sipsma et al., 2013 [47]	2007	Liberia	Population-based design	Demographic Health Survey	Cross-sectional	Multi-variable logistic regression (all forms of violence included within same model)	- Past 12-mo visit to health facility for self or own children	Upper-middle category
12	Sobkoviak, Yount, and Halim, 2012 [48]	2007	Liberia	Population-based design	Demographic Health Survey	Cross-sectional	Multi-variable linear or logistic regression (all forms of violence included within same model)	- Child’s weight-for-height (interval) - Child’s weight-for-age (interval) - Child’s height-for-age (interval) - Child’s z-scores were less than two SDs below the mean for wasted - Child’s z-scores were less than two SDs below the mean for underweight - Child’s z-scores were less than two SDs below the mean for stunted	Upper-middle category
13	Hayes and van Baak, 2017 [49]	2012–2013	Mali	Population-based design	Demographic Health Survey	Cross-sectional	Logistic regression	- Lifetime sexual IPV - Lifetime physical IPV	Upper-middle category
14	DeLong et al., 2020 [50]	2016	Niger	Other non-population-based design	RMA study	Cross-sectional (baseline)	Multi-variable logistic regression (violence typologies included within same model & separate models for girls 15–16 & 17–19 years old)	- Current use of modern family planning - Lifetime UIP experience	Upper-middle category
15	Silverman et al., 2020 [51]	2016	Niger	Intervention study	RMA study	Cross-sectional (baseline)	Multi-variable logistic regression (violence typologies included within same model)	- Current use of reversible modern family planning method	Upper-middle category
16	Kana, et al., 2020 [52]	2017–2019	Nigeria	Other non-population based design	Not stated	Cross-sectional	Multi-variable logistic regression	- Birth weight among term births	Top category
17	Abiodun et al., 2020 [53]	2019	Nigeria	Other non-population based design	not stated	Cross-sectional	Multi-variable logistic regression	- Past 12-mo experience of IPV	Top category
Southern and Eastern Africa									
18	Kidman, Palermo and Bertrand, 2015 [54]	2007	Democratic Republic of Congo	Population-based design	Demographic Health Survey	Cross-sectional	Multi-variable logistic regression (separate models for each form of IPV)	- Current use of modern contraception	Upper-middle category
19	Fleming et al., 2015 [55]	2009–2012	Democratic Republic of Congo ^+^	Intervention study	IMAGES	Cross-sectional (baseline)	Multi-variable logistic regression	- Lifetime physical IPV perpetration	Top category
20	Johnson et al., 2010 [56]	2010	Democratic Republic of Congo	Population-based design ^++^	Not stated	Cross-sectional	Bivariate comparisons of prevalence calculated using the adjusted Wald test of association (modeled for males and females)	- Substance abuse - Major depressive disorder - PTSD - Suicidal ideation - Suicide attempt	Upper-middle category
21	Gichane et al., 2018 [57]	2013–2014	Democratic Republic of Congo	Intervention study	Unnamed—Conditional Cash Transfer study	Longitudinal	Adjusted prevalence ratios (forms of violence included within separate models)	- Viral suppression at 6-week follow-up - Uptake of PMTCT services at 6-week follow up - ARV adherence measured at 6-week follow up	Top category
22	Tiruneh et al., 2018 [58]	2013–2014	Democratic Republic of the Congo	Population-based design	Demographic Health Survey	Cross-sectional	Multi-variable logistic regression (two models: one with physical, sexual, and emotional IPV as separate variables, and the second with any IPV) ^+++^	- Lifetime unintended pregnancy - Lifetime pregnancy loss - Lifetime physical IPV - Lifetime sexual IPV - Lifetime emotional IPV - Lifetime any IPV	Upper-middle category
23	Mpody et al., 2019 [59]	2016–2018	Democratic Republic of Congo	Intervention study	CQI-PMTCT study	Cross-sectional (baseline)	Multi-variable logistic regression (separate models based on pregnancy status during enrollment)	- Current presence of HBsAG in blood	Upper-middle category
24	Onsomu et al., 2015 [60]	2008–2009	Kenya	Population-based design	Demographic Health Survey	Cross-sectional	Multi-variable logistic regression (separate models for each form of IPV)	- HIV serostatus	Top category
25	Shi, Kouyoumdjian and Dushoff, 2013 [61]	2008–2009	Kenya	Population-based design	Demographic Health Survey	Cross-sectional	Generalized linear mixed models	- Current HIV^+^ status	Top category
26	Seff and Stark, 2019 [62]	2010	Kenya ^+^	Population-based design ^++^	Violence Against Children Surveys	Cross-sectional	Multi-variable logistic regression (separate models for males and females)	- Lifetime suicide ideation	Upper-middle category
27	Phillips-Howard et al., 2015 [63]	2011–2012	Kenya	Other non-population based design	Not stated	Cross-sectional	Multi-variable logistic regression (separate models for married and never-married women)	- Commercial pad use - Commercial pad provided by sexual partner	Upper-middle category
28	Kinuthia et al., 2018 [64]	2013	Kenya	Other non-population based design	Not stated	Cross-sectional	Multi-variable logistic regression	- Current non-disclosure of HIV- status	Top category
29	Goyette et al., 2018 [65]	2013–2015	Kenya	Intervention study	not stated	Longitudinal	Log-binomial generalized estimated equation	- Partner tested for HIV - Partner newly diagnosed with HIV - Partner new linkage to care for HIV	Upper-middle category
30	Wagman et al., 2016 [66]	1999–2006	Uganda	Intervention study	RCCS in Rakai	Longitudinal	Multi-variable linear regressions (all forms of violence included within same model and model includes an aggregate of each wave of data collection)	- Divorce or separation from husband or primary sexual partner	Upper-middle category
31	Kouyoumdjian et al., 2013 [67]	2000–2009	Uganda	Intervention study	RCCS in Rakai	Longitudinal	Multi-variable population attributable fraction (separate models for forms of IPV)	- Past-year HIV infection - Lifetime HIV infection	Top category
32	Speizer, 2010 [68]	2006	Uganda	Population-based design ^++^	Demographic Health Survey	Cross-sectional	Multi-variable multinomial logistic regression (separate models for married or unionized men and women)	- Attitudinal acceptance of violence	Upper-middle category
33	Macucha and Taunde, 2020 [69]	2016–2017	Mozambique	Retrospective case review	Not stated	Cross-sectional	Descriptive analysis of autopsy reports	- Homicide victimization	Upper-middle category
34	Adjiwan and N’Bouke, 2015 [70]	2010	Zimbabwe ^+^	Population-based design	Demographic Health Survey	Cross-sectional	Multi-variable probit model	- Current use of modern, non-permanent contraception	Upper-middle category
35	Shamu et al., 2016 [71]	2011	Zimbabwe	Other non-population based design	not stated	Cross-sectional	Multi-variable logistic regression (all forms of violence included within same model)	- Past 4-week depression - Lifetime suicidal thoughts	Upper-middle category
36	Shamu et al., 2018 [72]	2011	Zimbabwe	Other non-population based design	Not stated	Cross-sectional	Multi-variable logistic regression (all forms of violence included within same model)	- Lifetime history of miscarriage and/or stillbirth - Lifetime history of a miscarriage - Lifetime history of neonatal death - Lifetime history of a neonatal death, miscarriage, and/or stillbirth - History of an unplanned pregnancy - History of late booking into care - History of never booking into care - Recent negative birth outcome - Recent low birth weight	Upper-middle category
37	Nyamukoho et al., 2019 [73]	2016	Zimbabwe	Other non-population based design	Not stated	Cross sectional	Logistic regression	- Depression	Lower-middle category
Caribbean									
38	Kayibanda and Alary, 2020 [74]	2000, 2005, 2012	Haiti	Population-based design	Demographic Health Survey	Cross-sectional	Multi-variable analysis adjusted prevalence ratios (all forms of violence included within same model)	- Lifetime physical IPV perpetration	Upper-middle category
39	Gage, 2016 [75]	2013	Haiti	Intervention study ^++^	SAFE Dates	Cross-sectional (baseline)	Multi-variable logistic regression (both forms of violence included in model and separate models for males and females)	- Lifetime psychological IPV perpetration (ratio) - Lifetime physical/sexual IPV perpetration (ratio)	Upper-middle category
40	Saxena et al., 2019 [76]	2013	Haiti	Intervention study	GHESKIO	Cross-sectional (baseline)	Multi-variable logistic regression (all violence forms included within same model)	- Current HIV ^+^ status	Upper-middle category
41	Occean et al., 2020 [77]	2016–2017	Haiti	Population-based design	Demographic Health Survey	Cross-sectional	Multi-variable logistic regression	- Lifetime any IPV	Upper-middle category
42	Zalla et al., 2019 [78]	2016–2017	Haiti	Other non-population based design	Local AIDS Control Efforts (PLACE) study	Cross-sectional	Bivariate log-binomial regression (separate models for each group—MSM, FSW, TWG)	- Viral load from dried blood spots (ratio)	Upper-middle category
Central and Southeast Asia									
43	Bellizzi et al., 2019 [79]	2015	Afghanistan ^+^	Population-based design	Demographic Health Survey	Cross-sectional	Multi-variable logistic regression	- Past 3-year eclampsia around childbirth	Upper-middle category
44	Gibbs, Corboz, and Jewkes, 2018 [80]	2016–2017	Afghanistan	Intervention study	WfWI trial	Cross-sectional (baseline)	Multi-variable linear regressions (separate models for forms and rates of IPV)	- Past-week depression - Past-week PTSD symptoms - Current life satisfaction (ratio) - Lifetime suicidal ideation - Current overall health - Current functional limitations or disability (ratio)	Upper-middle category
45	Aye, et al., 2020 [81]	2016	Myanmar	Population-based design ^++^	Demographic Health Survey	Cross-sectional	Multi-variablelinear regression	- Mental distress	Top category
46	Tsai, Cappa and Petrowski, 2016 [82]	2013	Philippines	Population-based design	Demographic Health Survey	Cross-sectional	Multi-variable logistic regression (separate models for each form of IPV)	- Current use of contraception	Upper-middle category
47	Falb et al., 2014 [83]	2008	Thai-Myanmar Border	Other non-population based design	Reproductive Health Assessment Toolkit for Conflict-Affected women	Cross-sectional	Multi-variable generalized estimated equations	- Symptoms of pregnancy complications for the most recent pregnancy that resulted in a live birth in the last two years	Upper-middle category
48	Fellmeth et al., 2020 [84]	2015–2016	Thai-Myanmar Border	Other non-population based design	Not stated	Longitudinal	Multi-variable logistic regression	- Moderate or severe perinatal depression	Upper-middle category
Multiple Countries									
49	Gibbs et al., 2019 [85]	2016–2017 2017	Afghanistan Palestine	Afghanistan: Intervention study Palestine: Population-based design	Afghanistan: WfWI Palestine: not stated	Cross-sectional (baseline) Cross-sectional	Multi-variable logistic regression	- Family history of girl or woman relative being killed in the name of honor by another family member	Upper-middle category
50	Misch and Yount, 2014 [86]	2008–2009 2007 2010–2011	Kenya Liberia Zimbabwe ^+^	Population-based design	Demographic Health Survey	Cross-sectional	Multi-variable logistic regression (violence typologies included within same model)	- Previous birth breastfeeding initiation 1-h after delivery - Past 24-h exclusive breastfeeding	Upper-middle category
51	Gámez and Speizer, 2010 [87]	2007 2007 2005–2006	Liberia DRC Zimbabwe ^+^	Population-based design	Demographic Health Survey	Cross-sectional	Multi-variable logistic regression	- Premarital sexual debut	Upper-middle category

Note: When not explicitly indicated by the authors, the designation of “primary analysis model” was based on the first multivariate model provided per article. Thus, sensitivity testing was not included. If an article did not provide multivariate findings, then bivariate findings were reported; if bivariate findings were not reported, then descriptive findings were reported. ^+^ indicates that data from at least one other country was included in the article but excluded from this review; ^++^ indicates that the article includes data from men; ^+++^ indicates that two stages of analytical modeling were included for extraction; ^++++^ indicates that only non-binary measurements are indicated in parentheses. Acronyms—mo: month; HIV: Human Immunodeficiency Virus; ATI: Antiretroviral Therapy Interruption; IPV: intimate partner violence; MSM: Men who have Sex with Men; FSW: Female Sex Workers; TWG: Transgender Women; DRC: Democratic Republic of the Congo; PTSD: Post-Traumatic Stress Disorder; SDs: Standard Deviations; UIP: Unintended last Pregnancy; PMTCT: Prevention of Mother To Child Transmission; ARV: Antiretrovirals; HBsAG: Hepatitis-B Virus Surface Antigen; CQI-PMTCT: Continuous Quality Improvement for Prevention of Mother To Child Transmission; PLACE: Priorities for Local AIDS Control Efforts; WfWI: Women for Women International; RMA: Reaching Married Adolescents; IMAGES: International Men and Gender Equality Survey; GHESKIO: Haitian Group for the Study of Kaposi’s Sarcoma and Opportunistic Infections; RCCS: Rakai Community Cohort Study.

**Table 2:** Associations with intimate partner violence, per study.

Impact Categories, by Level of Ecological Framework	Corresponding Article																		
	1	2	3	4	5	6	7	8	9	10	11	12	13	14	15	16	17	18	19
Individual/survivor level																			
HIV and other sexually transmitted infections	-	-	-	-	-	-	-	-	-	-	-	-	-	-	-	-	-	-	-
Pregnancy, birth, and infancy	-	-	-	-	-	-	-	-	-	S	-	-	-	-	-	S	-	-	-
Substance use	-	-	-	-	-	-	-	-	-	-	-	-	-	-	-	-	-	-	-
Mental health	S	-	-	S	S	S	-	-	-	-	-	-	-	-	-	-	-	-	
Overall health	-	N/A	-	-	-	-	-	-	-	-	-	-	-	-	-	-	-	-	-
Fatal injuries	-	-	-	-	-	-	-	-	-	-	-	-	-	-	-	-	-	-	-
Reproductive health	-	-	S	-	-	-	-	-	-	-	-	-	-	S	S	-	-	S	-
Perpetration	-	-	-	-	-	-	-	-	-	-	-	-	-	-	-	-	-	-	-
Revictimization	-	-	-	-	-	-	-	-	-	-	-	-	-	-	-	-	S	-	-
Healthcare access, adherence, or disclosure	-	-	-	-	-	-	NS	-	-	-	S	-	-	-	-	-	-	-	-
Microsystem/relationship level																			
IPV perpetration during adulthood (children)	-	-	-	-	-	-	-	-	-	-	-	-	-	-	-	-	-	-	NS
Lifetime IPV victimization (children)	-	-	-	-	-	-	-	S	-	-	-	-	S	-	-	-	-	-	-
Physical health conditions (children)	-	-	-	-	-	-	-	-	-	-	-	S	-	-	-	-	-	-	-
Emotional/behavioral problems (children)	-	-	-	-	-	-	-	-	S	-	-	-	-	-	-	-	-	-	-
Accepting attitudes of IPV (children)	-	-	-	-	-	-	-	-	-	-	-	-	-	-	-	-	-	-	
Martial disruption (partners)	-	-	-	-	-	-	-	-	-	-	-	-	-	-	-	-	-	-	-
Fatal injuries (familial homicides)	-	-	-	-	-	-	-	-	-	-	-	-	-	-	-	-	-	-	-
**Impact Categories, by Level of Ecological Framework**	**Corresponding Article**																		
	**20**	**21**	**22 ^+^**	**23**	**24**	**25**	**26**	**28**	**29**	**30**	**31**	**32**	**33**	**34**	**35**	**36**	**37**	**38**	**39**
Individual/survivor level																			
HIV and other sexually transmitted infections	-	NS	-	S	S	S	-	-	-	-	S	-	-	-	-	-	-	-	-
Pregnancy, birth, and infancy	-	-	S	-	-	-	-	-	-	-	-	-	-	-	-	S	-	-	-
Substance use	NS	-	-	-	-	-	-	-	-	-	-	-	-	-	-	-	-	-	-
Mental health	S	-	-	-	-	-	S	-	-	-	-	-	-		S	-	S	-	-
Overall health	-	-	-	-	-	-	-	-	-	-	-	-	-	-	-	-	-	-	-
Fatal injuries	-	-	-	-	-	-	-	-	-	-	-	-	N/A	-	-	-	-	-	-
Reproductive health	-	-	-	-	-	-	-	-	-	-	-	-	-	NS	-	-	-	-	-
Perpetration	-	-	-	-	-	-	-	-	-	-	-	-	-	-	-	-	-	S	-
Revictimization	-	-	-	-	-	-	-	-	-	-	-	-	-	-	-	-	-	-	-
Healthcare access, adherence, or disclosure	-	NS	-	-	-	-	-	S	NS	-	-	-	-	-	-	-	-	-	-
Microsystem/relationship level																			
IPV perpetration during adulthood (children)	-	-	-	-	-	-	-	-	-	-	-	-	-	-	-	-	-	S	S
Lifetime IPV victimization (children)	-	-	S	-	-	-	-	-	-	-	-	-	-	-	-	-	-	-	-
Emotional/behavioral problems (children)	-	-	-	-	-	-	-	-	-	-	-	-	-	-	-	-	-	-	-
Physical health conditions (children)	-	-	-	-	-	-	-	-	-	-	-	-	-	-	-	-	-	-	-
Accepting attitudes of IPV (children)	-	-	-	-	-	-	-	-	-	-	-	S	-	-	-	-	-	-	-
Martial disruption (partners)	-	-	-	-	-	-	-	-	-	S	-	-	-	-	-	-	-	-	-
Fatal injuries (familial homicides)	-	-	-	-	-	-	-	-	-	-	-	-	-	-	-	-	-	-	-
**Impact Categories, by Level of Ecological Framework**	**Corresponding Article**																		
	**40**	**41**	**42**	**43**	**44**	**45**	**46**	**47**	**48**	**49**	**50**	**51**							
Individual/survivor level																			
HIV and other sexually transmitted infections	S	-	S	-	-	-	-	-	-	-	-	-							
Pregnancy, birth, and infancy	-	-	-	S	-	-	-	NS	-	-	S	-							
Substance use	-	-	-	-	-	-	-	-	-	-	-	-							
Mental health	-	-	-	-	S	S	-	-	S	-	-								
Overall health	-	-	-	-	S	-	-	-	-	-	-	-							
Fatal injuries	-	-	-	-	-	-	-	-	-	-	-	-							
Reproductive health	-	-	-	-	-	-	S	-	-	-	-	NS							
Perpetration	-	-	-	-	-	-	-	-	-	-	-	-							
Revictimization	-	-	-	-	-	-	-	-	-	-	-	-							
Healthcare access, adherence, or disclosure	-	-	-	-	-	-	-	-	-	-	-	-							
Microsystem/relationship level																			
IPV perpetration during adulthood (children)	-	-	-	-	-	-	-	-	-	-	-	-							
Lifetime IPV victimization (children)	-	S	-	-	-	-	-	-	-	-	-	-							
Emotional/behavioral problems (children)	-	-	-	-	-	-	-	-	-	-	-	-							
Physical health conditions (children)	-	-	-	-	-		-	-	-	-	-	-							
Accepting attitudes of IPV (children)	-	-	-	-	-	-	-	-	-	-	-	-							
Martial disruption (partners)	-	-	-	-	-	-	-	-	-	-	-	-							
Fatal injuries (familial homicides)	-	-	-	-	-	-	-	-	-	S	-	-							

Note: The health categories were informed by the World Health Organization’s classification of health outcomes associated with intimate and non-IPV (WHO, 2013). Other categories were designated based on the authors’ knowledge of violence literature and the general themes presented in the findings. Thus, the categories should only be viewed as representative of the included literature and were not meant to be an exhaustive list of potential categories associated with violence. S indicates that at least one of the intimate partner violence variables included within the primary model(s) was significantly associated with a dependent variable, NS indicates that none of the intimate partner violence variables were significantly associated with a dependent variable, N/A indicates that inferential statistics were not conducted,—indicates that outcome group was not included within the analytical modeling. Significance was determined by a *p*-value of <0.05; if no *p*-value was provided then adjusted odds ratio were considered significant if the confidence intervals were consistently greater than or less than 1.00. ^+^ indicates that two stages of analytical modeling were included for extraction. Acronyms: IPV—intimate partner violence; HIV—Human Immunodeficiency Virus.

**Table 3:** Classification of intimate partner violence as independent variable(s), per study.

IPV Variable Classification, by Level		Corresponding Article Number																			
		1	2	3	4	5	6	7	8	9	10	11	12	13	14	15	16	17	18	19	
Personal victimization of intimate partner violence																					
Timeframe	Lifetime	-	x	x	x	x	-	x	-	-	-	x	-	-	x	x	-	-	x	-	
	Current partnership	-	-	-	-	-	-	-	-	-	-	-	-	-	-	-	-	-	-	-	
	Previous partnership	-	-	-	-	-	-	-	-	-	-	-	-	-	-	-	-	x	-	-	
	Past 12-mo or past year	x	-	-	x	-	-	-	-	-	-	-	x	-	-	-	-	-	-	-	
	Past 3-months	-	-	-	-	-	x	-	-	-	-	-	-	-	-	-	-	-	-	-	
	Past 1-month	-	-	-	-	x	-	-	-	-	-	-	-	-	-	-	-	-	-	-	
	During pregnancy	-	-	-	-	-	-	-	-	-	x	-	-	-	-	-	x	-	-	-	
	Last incidence	-	-	-	-	-	-	-	-	-	-	-	-	-	-	-	-	-	-	-	
Forms	Multiple forms or any IPV	x	-	-	-	x	-	x	-	-	x	-	x	-	-	-	x	x	x	-	
	AGBH	-	-	-	-	-	-	-	-	-	-	-	-	-	-	-	-	-	-	-	
	Emotional	-	-	-	-	-	x	-	-	-	-	-	-	-	-	-	x	-	-	-	
	Physical	-	x	x	x	-	x	-	-	-	-	x	-	-	x	x	x	-	-	-	
	Physiological	-	-	x	x	-	-	-	-	-	-	-	-	-	-	-	-	-	-	-	
	Sexual	-	-	x	x	-	x	-	-	-	-	x	x	-	x	x	x	-	x	-	
	Verbal	-	-	-	-	-	-	-	-	-	-	-	-	-	-	-	-	-	-	-	
Measurement	Nominal (binary)	x	x	x	-	-	x	x	-	-	x	x	-	-	x	x	x	x	x	-	
	Nominal (>2 categories)	-	-	-	x	-	-	-	-	-	-	-	-	-	-	-	-	-	-	-	
	Ordinal	-	-	-	-	-	-	-	-	-	-	-	-	-	-	-	-	-	-	-	
	Interval	-	-	-	-	-	-	-	-	-	-	-	-	-	-	-	-	-	-	-	
	Ratio	-	-	-	-	x	-	-	-	-	-	-	-	-	-	-	-	-	-	-	
Childhood witnessing of intimate partner violence between parents																					
Timeframe	During childhood	-	-	-	-	-	-	-	x	x	-	-	x	x	-	-	-	-	-	x	
Forms	Multiple forms or any IPV	-	-	-	-	-	-	-	x	x	-	-	x	x	-	-	-	-	-	x	
	Physical	-	-	-	-	-	-	-	-	-	-	-	-	-	-	-	-	-	-	-	
Measurement	Nominal (binary)	-	-	-	-	-	-	-	x	x	-	-	x	x	-	-	-	-	-	x	
Community rates of intimate partner violence																					
Timeframe	Lifetime of respondents	-	-	-	-	-	-	-	-	-	-	-	-	-	-	-	-	-	-	-	
	Past 5-years	-	-	-	-	-	-	-	-	-	-	-	-	-	-	-	-	-	x	-	
Forms	Multiple forms	-	-	-	-	-	-	-	-	-	-	-	-	-	-	-	-	-	x	-	
	Physical	-	-	-	-	-	-	-	-	-	-	-	-	-	-	-	-	-	-	-	
	Sexual	-	-	-	-	-	-	-	-	-	-	-	-	-	-	-	-	-	x	-	
Measurement	Nominal (binary)	-	-	-	-	-	-	-	-	-	-	-	-	-	-	-	-	-	-	-	
	Ordinal	-	-	-	-	-	-	-	-	-	-	-	-	-	-	-	-	-	-	-	
	Ratio	-	-	-	-	-	-	-	-	-	-	-	-	-	-	-	-	-	x	-	
**IPV Variable Classification, by Level**		**Corresponding Article Number**																			
		**20**	**21**	**22**	**23**	**24**	**25**	**26**	**27**	**28**	**29**	**30 ^+^**	**31**	**32**	**33**	**34**	**35**	**36**	**37**	**38**	**39**
Personal victimization of intimate partner violence																					
Timeframe	Lifetime	x	x	x	x	x	x	x	x	-	x	-	x	-	-	-	x	x	x	x	-
	Current partnership	-	-	-	-	-	-	-	-	x	-	-	-	-	-	x	-	-	-	-	-
	Previous partnership	-	-	-	-	-	-	-	-	-	-	-	-	-	-	-	-	-	-	-	-
	Past 12-mo or past year	-	-	-	-	-	-	-	-	-	-	x	-	-	-	-	-	x	-	-	-
	Past 3-months	-	-	-	-	-	-	-	-	-	-	-	-	-	-	-	-	-	-	-	-
	Past 1-month	-	-	-	-	-	-	-	-	-	-	-	-	-	-	-	-	-	-	-	-
	During pregnancy	-	-	-	-	-	-	-	-	-	-	-	-	-	-	-	-	x	-	-	-
	Last incidence	-	-	-	-	-	-	-	-	-	-	-	-	-	x	-	-	-	-	-	-
Forms	Multiple forms or any IPV	x	x	x	x	x	x	x	x	x	x	-	x	-	x	x	x	x	x	x	-
	AGBH	-	-	-	-	x	-	-	-	-	-	-	-	-	-	-	-	-	-	-	-
	Emotional	-	x	x	-	x	-	-	-	-	-	x	-	-	-	-	x	x	-	-	-
	Physical	-	x	x	-	x	-	-	-	-	-	x	x	-	-	-	x	x	-	-	-
	Physiological	-	-	-	-	-	-	-	-	-	-	-	-	-	-	-	-	-	-	-	-
	Sexual	-	x	x	-	x	-	-	-	-	-	x	x	-	-	-	x	x	-	-	-
	Verbal	-	-	-	-	-	-	-	-	-	-	-	x	-	-	-	-	-	-	-	-
Measurement	Nominal (binary)	x	x	x	x	x	-	x	x	x	x	x	x	-	x	x	-	x	x	x	-
	Nominal (>2 categories)	-	-	-	-	-	-	-	-	-	-	-	-	-	-	-	-	-	-	-	-
	Ordinal	-	-	-	-	-	-	-	-	-	-	-	x	-	-	-	x	-	-	-	-
	Interval	-	-	-	-	-	x	-	-	-	-	-	-	-	-	-	-	-	-	-	-
	Ratio	-	-	-	-	-	-	-	-	-	-	-	-	-	-	-	-	-	-	-	-
Childhood witnessing of intimate partner violence between parents																					
Timeframe	Lifetime of respondents	-	-	x	-	-	-	-	-	-	-	-	-	x	-	-	-	-	-	x	x
Forms	Multiple forms or any IPV	-	-	x	-	-	-	-	-	-	-	-	-	x	-	-	-	-	-	x	x
	Physical	-	-	-	-	-	-	-	-	-	-	-	-	-	-	-	-	-	-	-	-
Measurement	Nominal (binary)	-	-	x	-	-	-	-	-	-	-	-	-	x	-	-	-	-	-	x	x
Community rates of intimate partner violence																					
Timeframe	Lifetime of respondents	-	-	x	-	-	-	-	-	-	-	-	-	-	-	-	-	-	-	-	-
	Past 5-years	-	-	-	-	-	-	-	-	-	-	-	-	-	-	-	-	-	-	-	-
Forms	Multiple forms	-	-	x	-	-	-	-	-	-	-	-	-	-	-	-	-	-	-	-	-
	Physical	-	-	-	-	-	-	-	-	-	-	-	-	-	-	-	-	-	-	-	-
	Sexual	-	-	-	-	-	-	-	-	-	-	-	-	-	-	-	-	-	-	-	-
Measurement	Nominal (binary)	-	-	-	-	-	-	-	-	-	-	-	-	-	-	-	-	-	-	-	-
	Ordinal	-	-	x	-	-	-	-	-	-	-	-	-	-	-	-	-	-	-	-	-
	Ratio	-	-	-	-	-	-	-	-	-	-	-	-	-	-	-	-	-	-	-	-
**IPV Variable Classification, by Level**		**Corresponding Article Number**																			
		**40**	**41**	**42**	**43**	**44**	**45**	**46**	**47**	**48**	**49**	**50**	**51**								
Personal victimization of intimate partner violence																					
Timeframe	Lifetime	x	-	-	x	x	x	-	x	x	-	-	-								
	Current partnership	-	-	-	-	-	-	-	-	-	-	-	-								
	Previous partnership	-	-	-	-	-	-	-	-	-	-	-	-								
	Past 12-mo or past year	-	-	x	-	-	x	x	-	-	x	-	-								
	Past 3-months	-	-	-	-	-	-	-	-	-	-	-	-								
	Past 1-month	-	-	-	-	-	-	-	-	-	-	-	-								
	During pregnancy	-	-	-	-	-	-	-	-	-	-	x	-								
	Last incidence	-	-	-	-	-	-	-	-	-	-	-	-								
Forms	Multiple forms or any IPV	-	-	-	-	-	x	-	x	x	-	-	-								
	AGBH	-	-	-	-	-	-	-	-	-	-	-	-								
	Emotional	-	-	-	-	x	x	x	-	-	-	x	-								
	Physical	x	-	x	-	x	x	x	-	-	x	x	-								
	Physiological	x	-	-	-	-	-	-	-	-	-	-	-								
	Sexual	x	-	-	x	-	x	x	-	-	-	x	-								
	Verbal	-	-	-	-	-	-	-	-	-	-	-	-								
Measurement	Nominal (binary)	x	-	x	x	-	x	x	x	x	x	x	-								
	Nominal (>2 categories)	-	-	-	-	-	-	-	-	-	-	-	-								
	Ordinal	-	-	-	-	x	-	-	-	-	-	-	-								
	Interval	-	-	-	-	-	-	-	-	-	-	-	-								
	Ratio	-	-	-	-	-	-	-	-	-	-	-	-								
Childhood witnessing of intimate partner violence between parents																					
Timeframe	Lifetime of respondents	-	x	-	-	-	-	-	-	-	-	-	-								
Forms	Multiple forms or any IPV	-	x	-	-	-	-	-	-	-	-	-	-								
	Physical	-	-	-	-	-	-	-	-	-	-	-	-								
Measurement	Nominal (binary)	-	x	-	-	-	-	-	-	-	-	-	-								
Community rates of intimate partner violence																					
Timeframe	Lifetime of respondents	-	-	-	-	-	-	-	-	-	-	-	x								
	Past 5-years	-	-	-	-	-	-	-	-	-	-	-	-								
Forms	Multiple forms	-	-	-	-	-	-	-	-	-	-	-	x								
	Physical	-	-	-	-	-	-	-	-	-	-	-	-								
	Sexual	-	-	-	-	-	-	-	-	-	-	-	-								
Measurement	Nominal (binary)	-	-	-	-	-	-	-	-	-	-	-	-								
	Ordinal	-	-	-	-	-	-	-	-	-	-	-	-								
	Ratio	-	-	-	-	-	-	-	-	-	-	-	x								

Note: ^+^ indicates that multiple forms of IPV were included in study but not all IPV variables were included in inferential model. Acronyms—mo: month; IPV: intimate partner violence; AGBH: aggravated bodily harm.

**Table 4:** AXIS quality assessment of included articles, frequency per item, and quartile distribution of final score.

	n	%
Introduction		
1. Were the aims/objectives of the study clear?	51	100.00%
Methods		
2. Was the study design appropriate for the stated aim(s)? ^+^	45	88.24%
3. Was the sample size justified?	28	54.90%
4. Was the target/reference population clearly defined? (Is it clear who the research was about?)	51	100.00%
5. Was the sample frame taken from an appropriate population base so that it closely represented the target/reference population under investigation?	41	80.39%
6. Was the selection process likely to select subjects/participants that were representative of the target/reference population under investigation?	45	88.24%
7. Were measures undertaken to address and categorize non-responders?	23	45.10%
*7.a Were measures undertaken to categories non-responders (i.e., do the authors* *identify the non-response rate)?*	*43*	*84.31%*
*7.b Were measures undertaken to address* *non-responders (i.e., do the authors identify how the non-response rate was addressed)?*	*25*	*49.02%*
8. Were the risk factor and outcome variables measured appropriately to the aims of the study?	25	49.02%
9. Were the risk factor and outcome variables measured correctly using instruments/measurements that had been trialed, piloted, or published previously?	13	25.49%
*9.a Were the independent IPV variables measured correctly using instruments/* *measurements that had been trialed, piloted, or published previously?*	*24*	*47.06%*
*9.b Were the outcome variables measured correctly using instruments/* *measurements that had been trialed, piloted, or published previously?*	*18*	*35.29%*
10. Is it clear what was used to determined statistical significance and/or precision estimates (e.g., *p* values, CIs) ?	50	98.04%
*10.a Was sensitivity testing conducted?*	*2*	*3.92%*
11. Were the methods (including statistical methods) sufficiently described to enable them to be repeated?	37	72.55%
Results		
12. Were the basic data adequately described?	49	96.08%
13. Does the response rate raise concerns about non-response bias? ^+,++^	2	3.92%
14. If appropriate, was information about non-responders described? ^+^	1	1.96%
15. Were the results internally consistent?	28	54.90%
*15.a Were bivariate and multivariate analyses available?* ^+++^	*33*	*64.71%*
16. Were the results for the analyses described in the methods, presented?	50	98.04%
Discussion		
17. Were the authors’ discussions and conclusions justified by the results?	47	92.16%
18. Were the limitations of the study discussed?	49	96.09%
Other		
19. Were there any funding sources or conflicts of interest that may affect the authors’ interpretation of the results? ^++^	23	45.10%
20. Was ethical approval or consent of participants attained?	35	68.63%

Note: Quartile distribution was as follows: 31.37% (n = 16) top category, 62.75% (n = 32) upper-middle category, 5.88% (n = 3) lower-middle category, 0.00 % (n = 0) lowest category. ^+^ indicates an item where only one reviewer’s votes were applied; ^++^ indicates that consistent “No” voting were applied to these articles; ^+++^ descriptive studies were not included in the % calculation (n = 49). Acronyms—IPV: intimate partner violence. Notations—%: percentage of included studies; n: number of studies. For AXIS items that had two separate components (e.g., items 7 and 9), reviewers voted for each component separately; only articles that reviewed affirmative votes for both components by both reviewers were counted for the corresponding item. Information it *italics* represents additional considerations, beyond the AXIS designations. While not impacting the final score, two additional sub-items were included to determine the frequency that articles included sensitivity testing (*10.a*) or provided both bivariate and multivariate results (*15.a*).

## Data Availability

Not applicable.
